# Evaluation of previous management against a developed clinical pathway for chronic rhinosinusitis with nasal polyposis in Universiti Kebangsaan Malaysia Medical Center

**DOI:** 10.1097/MD.0000000000027675

**Published:** 2021-11-05

**Authors:** Timothy LW Wong, Salina Husain, Aniza Ismail, Farah Dayana Zahedi, Syed Mohamed Aljunid, Amrizal Muhammad Nur

**Affiliations:** a Department of Otorhinolaryngology-Head and Neck Surgery, Universiti Kebangsaan Malaysia Medical Center, Jalan Yaacob Latif, Bandar Tun Razak, Cheras, Kuala Lumpur, Malaysia; b Surgical Based Department, Faculty of Medicine and Health Sciences, Universiti Malaysia Sabah, Kota Kinabalu, Sabah, Malaysia; c Department of Community Health, Universiti Kebangsaan Malaysia Medical Center, Jalan Yaacob Latif, Bandar Tun Razak, Cheras, Kuala Lumpur, Malaysia; d International Center for Casemix and Clinical Coding, Faculty of Medicine, Universiti Kebangsaan Malaysia; e Department of Health Policy and Management, Faculty of Public Health, Kuwait University, Kuwait.

**Keywords:** chronic rhinosinusitis, clinical pathway, nasal polyp

## Abstract

The study aims to evaluate previous management of CRSwNP patients in Universiti Kebangsaan Malaysia Medical Center (UKMMC) against a developed CP.

Chronic rhinosinusitis with nasal polyposis (CRSwNP) has high economic burden and impacts patient's quality of life. Implementation of clinical pathway (CP) can standardize care while optimizing resources.

Analytical cross-sectional

This study utilized medical records of 103 CRSwNP patients at UKMMC otorhinolaryngology clinic from 2010 to 2015. Patients were divided into groups who underwent or did not undergo surgery. Information was obtained regarding sociodemographic, follow-ups, pharmaceutical regimes, and treatment cost. Cost analysis was done using top-down analysis and activity-based costing and CP was formulated. Cost was calculated using year 2020 rates to adjust for inflation. (United States Dollars [USD]1 = Ringgit Malaysia [RM] 4.2015)

Study showed non-CP patients were undertreated compared to CP. This affects clinical outcomes as optimal treatment demanded by CP was not achieved. Total cost for non-CP, non-surgery patients were lower (USD660) compared to CP (USD780) due to under treatment and shorter follow-ups. Meanwhile, total cost for non-CP surgery patients were higher (USD3600) compared to CP (USD2706) due to longer visit durations and hospital stays. Non-CP surgery group underwent lengthy follow-up duration (20.7 months) prior to operation compared to 12 months expected in CP.

Study showed non-CP patients were undertreated compared to CP. We identified aspects which resulted in resource wastage and unnecessary burden to our healthcare system. This study enables development of a written CP by fine-tuning various aspects of CP which could be applied to our future practice.

## 1 Introduction

Clinical pathway (CP) is a multidisciplinary plan of care based on best clinical practice for specified groups of patients with a particular diagnosis designed to minimize delays, optimize resource utilization and maximize quality of care.^[1,2]^ In short, the aim is for patients to achieve predetermined outcomes within a specified time frame. With an ever-increasing population, countries worldwide have started to scrutinize their healthcare policies to emphasize on cost effective practices while still improving on quality of patient care.^[3]^

Not all cases are suitable to be implemented in CP form. In general, CP concentrates on cases with high volume, high-cost diagnoses and procedures in which CP can have maximal impact in terms of cost and quality of care. There should be good response and interest from all medical staffs, those where variations in practice occur and affect patient outcome. For most medical diagnoses, CP is difficult to translate successfully in care of patients due to greater heterogeneity among patients and multiple comorbidities.^[4]^

The benefits of CP are the introduction of evidence-based medicine and use of clinic guidelines, optimization of resources, reduction of variances in patient care by promoting standardization, improvement of clinical outcomes, support for clinical audit and risk management, improvement of multi-disciplinary communication, teamwork and care planning as well as improving and reducing patient documentation.

Chronic rhinosinusitis (CRS) is inflammation of nasal mucosa and paranasal sinuses for more than 12 consecutive weeks.^[5]^ It is classified into chronic rhinosinusitis without nasal polyposis and chronic rhinosinusitis with nasal polyposis (CRSwNP). Diagnosis of CRS is based on clinical symptoms and either endoscopic signs and/or computed tomography (CT) scan findings.^[6]^

CRS is a common disease worldwide with reported prevalence rate ranging from 6% to 15%. The prevalence rate of CRS in Europe, United States and Brazil is between 5% to 15%.^[6]^ In Asian region, prevalence rate reported in Korea, China and Singapore are 7%, 8%, and 2.7% respectively.^[7]^ CRSwNP is associated with a prevalence of 2.5 ± 0.2% (mean ± standard error [SE]).^[8]^

The direct cost of CRS to society have been well documented and as of 2017, Rudmik et al reported the direct cost of CRS to be between $10 and $13 billion in the United States.^[9]^ A previous study done by Bhattacharyya et al had quantified the incremental per-patient, per-year health care cost burdens of CRS at $772.^[10]^ If CRS is severe to consider for surgery, the yearly cost related to CRS is even higher at $2449.^[11]^ The diagnosis and follow-up also involve expensive diagnostic procedures such as CT scans, cultures, and endoscopies. Antibiotic cost alone for CRS cost $150 million a year.^[12]^ Bhattacharyya et al estimated an average cost of $7726 for endoscopic sinus surgery and procedure-related follow-up (2 postoperative visits).^[11]^ CRS has been shown to cause significant activity, work, and social limitations.^[13]^ For an individual with medically refractory CRS, this represents a cost of $10,077 per year and nationally costs the United States an additional $12.8 billion.^[14]^

## 2 Justification of research

We aim to develop a CP for CRSwNP patients to improve quality of care while ensuring resource optimization. If proven effective, CP can minimize delays, improve clinical outcomes, and promote standardization. This study also identifies clinical variances in previous management and helps determine if our developed CP is acceptable or needs further improvement.

## 3 Materials and methodology

This analytical cross-sectional study was conducted at Universiti Kebangsaan Malaysia Medical Center (UKMMC) and was approved by UKM Ethics Committee. This approval encompasses clinical related aspects of the study. The confidentiality of all data was maintained. This study was conducted from July 2018 to December 2019. The study population include patients aged 18 years old and above diagnosed with CRSwNP seen in UKMMC otorhinolaryngology clinic from 2010 to 2015. The minimum sample size of 100 with power of 80% is calculated with a 95% confidence interval using Kish (1965) method. Sample size calculation was based on mean prevalence of CRSwNP of 7% in Korean population.^[7]^ No local study was done before and Korean study's mean prevalence was taken as it is based on an Asian population.

Medical records of CRSwNP patients were obtained via case mix system. Information from date of first diagnosis and subsequent follow-ups were collected using CP data collection sheet. The patients were divided into those who underwent surgery and those who did not undergo surgery. Data for cost analysis was requested from hospital's accounts department, clinic, and pharmacy. Top-down costing and activity-based costing data was obtained from UKMMC's International Center for Case Mix and Clinical Coding (ITCC). The cost of medication, surgery and hospital stay were calculated taking into account adjusted year 2020 level when the study was completed to reflect inflation. The prices of drugs, operations and materials have been constant barring inflation. The exchange rate was valued at year 2020 average level of United States Dollar (USD)1 to RM4.2015. This was used to calculate cost for patients who underwent or did not undergo surgery. Other information obtained were sociodemographic data, duration of follow-ups, pharmaceutical regimes given and outcomes in terms of nasal polyp resolution. These data were then compared with those of CP.

### 3.1 Statistical analysis

Statistical analysis was performed using Statistical Package for Social Sciences version 23.0. Means ± standard deviations were used to describe numerical variables, while frequencies and percentages the categorical ones. The normal distribution was checked with the Shapiro–Wilk test and data was presented as mean + standard deviation. Descriptive statistics was used to present demographics, medications given, polyps grading as well as cost for surgery and non-surgery patients. Data was studied to understand the significant differences between non-CP group and CP. The relationship between categorical variables were determined using Pearson Chi-Squared statistics and Fisher exact test when the single cell is relatively small. The associations between categorical independent variables and numerical dependent variables were evaluated using Independent Sample *t*-tests. The significant test for benchmarking fixed value was conducted using One Sample *t*-tests. The level of statistical significance was set at 0.05.

### 3.2 Inclusion criteria

The inclusion criteria for this study include CRSwNP patients aged 18 years old and above seen in UKMMC otorhinolaryngology clinic from 2010 to 2015. CRSwNP patients who have undergone prior functional endoscopic sinus surgery (FESS), or nasal polypectomy were also included in the study.

### 3.3 Exclusion criteria

Exclusion criteria consist of patients with sinonasal pathologies such as tumors, autoimmune nasal conditions, and granulomatous diseases. This also include patients who have undergone advanced endoscopic or open nasal surgeries for sinonasal malignancies, patients with other non- ear, nose, and throat related cancers and pregnant women. Patients who were involved in other clinical studies were excluded as well as those with missing data in their clinical records.

## 4 Results

### 4.1 Development of clinical pathway

**Figure 1:**
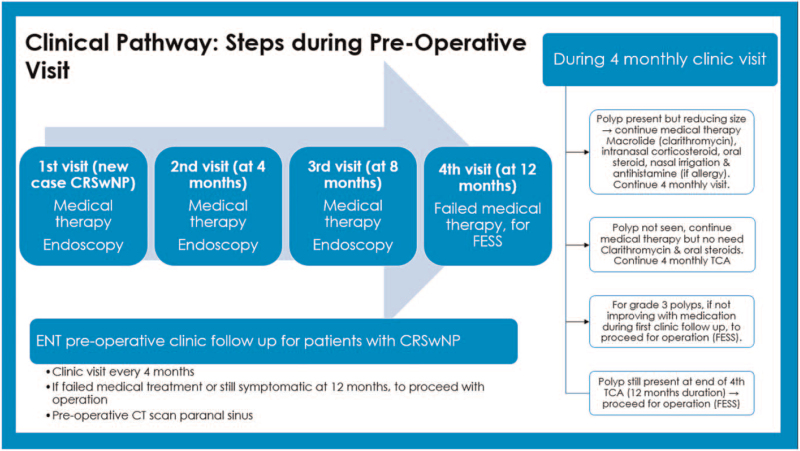
Clinical pathway: steps during preoperative visit. (FESS = functional endoscopic sinus surgery, CRSwNP = chronic rhinosinusitis with nasal polyposis, CT = computed tomography, TCA = to see again/ follow-up).

**Figure 2:**
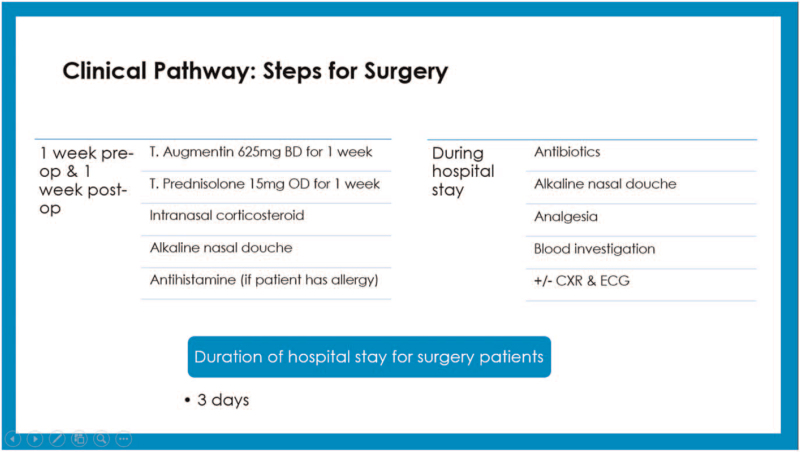
Clinical pathway: steps for surgery. (T. = tablet, BD = twice daily, OD = once daily, CXR = chest X-ray, ECG = electrocardiogram).

**Figure 3:**
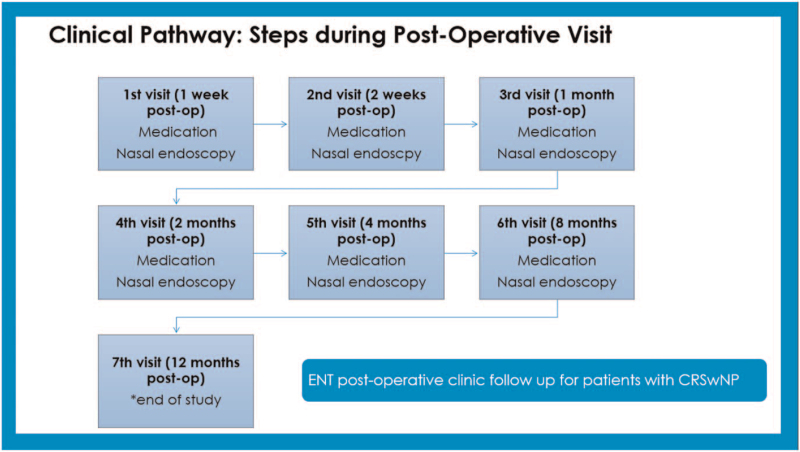
Clinical pathway: steps during postoperative visit. (ENT = ear, nose, and throat, CRSwNP = chronic rhinosinustis with nasal polyposis).

**Figure 4:**
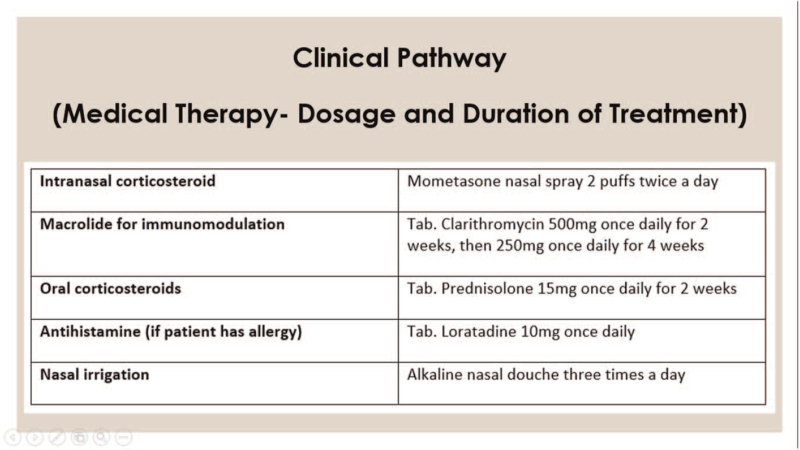
Clinical pathway: medical therapy – dosage and duration of treatment. (Tab = tablet).

CP development consisted of 2 phases. Phase one is development of CP by experts while phase 2 was retrospective data collection of non-CP patient group to compare with the developed CP. Phase one was based on discussion by experts in the field to develop a CP for management of CRSwNP in UKMMC which involved otolaryngologists specialising in field of rhinology and public health specialists with expertise in CP, cost analysis and quality of care. This CP is based on evidence-based medicine, value-based medicine, and clinical practice guidelines. It maps out the whole spectrum of long-term care provided to CRSwNP patients. This developed CP is used for comparison with practices done previously in non-CP patients from 2010 to 2015. The CP is divided into steps for non-surgery/ pre-operative visits, surgery, and post-operative visits (Figs. 1–4).

### 4.2 Demographic data

This study involved 103 CRSwNP patients of which 49 (47.6%) underwent surgery while 54 (52.4%) did not undergo surgery. For surgery group, mean age was 47.6 years old at first clinic visit while mean age for non-surgery patients was 59.9 years old. This is statistically significant as shown by *P* value of .000 (*P* < .05). Meanwhile, mean age of patients at surgery was 49.5 years old. For patients who underwent surgery, pre-operative clinic visit interval is 2.1 months while for non-surgery patients, mean clinic visit interval was 3 months. This showed a statistically significant *P* value of .000 (*P* < .05). This shows that patients who underwent surgery had more frequent clinic visits compared to those who did not undergo surgery. Mean total duration of clinic visits for non-surgery patients was 15 months while mean duration of clinic visits until operation date for surgery patients was 20.7 months. Patients who underwent surgery stayed in the ward for a mean duration of 4 days (Table 1).

**Table 1:** Age of 1st clinic visit, age of surgery, clinic visit interval, duration of clinic visits and duration of hospital stay.

	Surgery		Non-surgery		Total		
	Mean	SD	Mean	SD	Mean	SD	Significance (*P* value)
Age at 1st clinic visit	47.6	14.3	59.9	10.5	54.0	13.9	**<.001** ^∗^
Age at Surgery	49.5	14.9	.	.	49.5	14.9	.
Preop/ non-surgery clinic visit interval (mo)	2.1	0.8	3.0	0.8	2.5	0.9	**<.001** ^∗^
Total duration visits non-surgery (mo)	.	.	14.9	1.8	14.9	1.8	.
Duration 1st clinic visit until operation (mo)	20.7	21.7	.	.	20.7	21.7	.
Duration of hospital stay (d)	4.0	0.6	.	.	4.0	0.6	.

SD = standard deviation.

∗ Significant *P* value <.05.

68 (66%) CRSwNP patients were male while 35 (34%) were female. This ratio was approximately similar in groups who underwent surgery or did not undergo surgery. In surgery group, the highest proportion of patients were of Malay race at 28 (57.1%) patients while in non-surgery group, it was Chinese at 27 (50%) patients. This data is significant with a *P* value of .009 (*P* < .05).

Majority of CRSwNP patients, 75 (72.8%) were non-asthmatics, 56 (54.4%) had allergy to either food or medication and 58 (56.3%) had allergic rhinitis. There was no significance in relation of asthma, allergy, or allergic rhinitis to surgery status in patients as shown by *P* value of *P* > .05.

During first clinic visit, patients’ left, and right nostrils were assessed for polyps and the larger polyp out of the 2 nostrils was used to represent initial polyp size at presentation to standardize the data. It is of significance that in surgery patients, 45 (91.9%) had higher grade of grade 2 and 3 polyps. As for non-surgery patients, 45 (83.3%) had lower grade 1 and 2 polyp at first clinic visit. This is of statistical significance with *P* value of .000 (*P* < .05).

In terms of 6 months postoperation polyp recurrence, 27 (55.1%) patients had polyp recurrence. However, the majority 22 (44.9%) patients had small grade 1 polyp while 22 (44.9%) patients had no polyp recurrence. As for non-surgery patients, they were assessed based on whether there was resolution of nasal polyps at the end of 12 months of medical treatment. Majority of non-surgery patients, 48 (88.9%) patients still had nasal polyps at the end of the 12 months with total 87.1% having grade 1 and 2 polyps (Table 2).

**Table 2:** Demographic data, comorbidities, and nasal polyp size.

		Surgery		Non-surgery		Total		
		n	%	n	%	n	%	Significance (*P* value)
Gender	Female	17	34.7	18	33.3	35	34.0	.88
	Male	32	65.3	36	66.7	68	66.0	
Race	Malay	28	57.1	15	27.8	43	41.7	**.009** ^∗^
	Chinese	13	26.5	27	50.0	40	38.8	
	Indian	8	16.3	12	22.2	20	19.4	
Asthma	Yes	14	28.6	14	25.9	28	27.2	.76
	No	35	71.4	40	74.1	75	72.8	
Allergy	Yes	25	51.0	31	57.4	56	54.4	.52
	No	24	49.0	23	42.6	47	45.6	
Allergic rhinitis	Yes	31	63.3	27	50.0	58	56.3	.18
	No	18	36.7	27	50.0	45	43.7	
Polyp grade at first clinic visit	1	4	8.2	23	42.6	27	26.2	**<.001** ^∗^
	2	21	42.9	22	40.7	43	41.7	
	3	24	49.0	9	16.7	33	32.0	
Post-op 6 mo polyp recurrence	Yes	27	55.1	0	0.0	27	55.1	–
	No	22	44.9	0	0.0	22	44.9	
Polyp size at 6 mo postop	0	22	44.9	0	0.0	22	44.9	–
	1	22	44.9	0	0.0	22	44.9	
	2	4	8.2	0	0.0	4	8.2	
	3	1	2.0	0	0.0	1	2.0	
Non-surgery polyp resolved at 12 mo	Yes	0	0.0	6	11.1	6	11.1	-
	No	0	0.0	48	88.9	48	88.9	
Non-surgery polyp size at 12 mo	0	0	0.0	6	11.1	6	11.1	–
	1	0	0.0	30	55.6	30	55.6	
	2	0	0.0	17	31.5	17	31.5	
	3	0	0.0	1	1.9	1	1.9	

n = number.

∗ Significant *P* value <.05.

The frequency of medications prescribed during clinic visits were analyzed. Result revealed CRSwNP patients were prescribed clarithromycin in only 36.5% of clinic visits. Mean duration of clarithromycin prescribed is 25.5 days whereas CP requires 42 days. Clarithromycin regime is only given correctly 23.8% of the time (CP: 500 mg once daily for 2 weeks, then 250 mg once daily for 4 weeks). This showed patients are being undertreated compared to CP.

As for intranasal corticosteroid (INCS), it was prescribed during 95.6% of clinic visits for surgery and non-surgery groups. Based on CP, all CRSwNP patients should be prescribed dosage of 2 nasal sprays in each nostril twice daily. However, this is only prescribed in 62.7% of visits. From the findings, it also showed that INCS is only given in 91.9% of all clinic visits in surgery group compared to 99% in non-surgery group. This is statistically significant with a *p*-value of 0.015 (*P* < .05). The tendency to prescribe dosage of 2 INCS sprays twice a day is higher in surgery group at 72.9% compared to 53.4% in non-surgery group. This result is significant with a *P* value of .013 (*P* < .05). The non-surgery group of patients tend to get a reduced dose of INCS compared to the surgery group.

Data also showed oral corticosteroid was only given in 20% of all clinic visits with a mean duration of 7.76 days. For the surgery group, oral steroid was given in 25.2% of clinic visits while for non-surgery group, it was 15.3% of visits. This showed a statistical significance with *P* value of.02 (*P* < .05). Based on CP, patients should be given 14 days course of 15 mg daily oral corticosteroids at every clinic visit except for patients with contraindications. Result also revealed that nasal irrigation was given in only 15.6% of all clinic visits. Based on CP, patients should always be prescribed nasal irrigation. As for antihistamines, results showed that antihistamine was given in 68.2% of all clinic visits (Table 3).

**Table 3:** Percentage of times medication prescribed during clinical visits.

	Surgery		Non-surgery		Total		
Time Medication Given	Mean	SD	Mean	SD	Mean	SD	Significance (*P* value)
Clarithromycin (%)	39.5	27.9	33.9	22.5	36.5	25.2	.26
Clarithromycin duration (d)	23.7	12.9	27.1	15.2	25.5	14.2	.22
Correct Clarithromycin regime (%)	21.7	34.3	25.8	37.0	23.8	35.6	.57
Intranasal corticosteroid (%)	91.9	19.0	99.0	5.9	95.6	14.1	**.02** ^∗^
Two nasal sprays once daily (%)	23.8	36.0	46.0	41.1	35.4	40.2	**<.001** ^∗^
Two nasal sprays twice daily (%)	72.9	37.5	53.4	40.7	62.7	40.2	**.01** ^∗^
Oral corticosteroid (%)	25.2	23.9	15.3	17.7	20.0	21.4	**.02** ^∗^
Oral corticosteroid duration (d)	8.8	6.3	6.9	7.2	7.8	6.8	.16
Nasal irrigation (%)	18.0	22.2	13.4	21.6	15.6	21.9	.30
Antihistamine (%)	63.4	32.5	72.6	31.7	68.2	32.3	.15

SD = standard deviation.

∗ Significant *P* value <.05.

Cost analysis of patients who underwent surgery revealed that all non-CP pre-operative surgery cost was lower compared to that of CP in terms of minimum, maximum, average, and total visit cost. All these parameters showed significant *P* value of *P* < .05. However, surgery cost is higher in non-CP group at USD2004.50 compared to USD1526.50 in CP with significant *P* value of .00 (*P* < .05).

As for postoperation, all the non-CP post-operative cost comprising minimum, maximum, average, and total visit cost were higher than that of CP and all showed significant *P* value of *P* < .05 except for the minimum visit cost. In total, when all preoperative visit, surgery and postoperative visit costs were combined, result showed that cumulative cost for non-CP surgery patient is USD3600.20 which is significantly higher than that of CP at USD2706 (Table 4).

**Table 4:** Cost analysis for surgery patients.

Cost	CP	SD	Non-CP	SD	Significance (*P* value <.05)
Minimum preoperative visit (USD)	189.1	–	79.6		**<.001** ^∗^
Maximum preoperative visit (USD)	305.7	–	224.0		**<.001** ^∗^
Average pre-operative visit (USD)	229.3	61.6	139.9	24.7	**<.001** ^∗^
Total preoperative visit (USD)	688.0	4.8	1048.2	798.8	**.003** ^∗^
Surgery cost (USD)	1526.5	191.9	2004.5	222.4	**<.001** ^∗^
Minimum postoperative visit (USD)	55.5	–	56.9		.07
Maximum postoperative visit (USD)	114.3	–	141.6		**.001** ^∗^
Average post-operative visit (USD)	81.9	26.5	100.2	56.3	**.03** ^∗^
Total postoperative visit (USD)	491.5	4.7	547.5	134.2	**.005** ^∗^
Total cost for surgery group (USD)	2706.0		3600.2		

USD = US Dollars, CP = clinical pathway, SD = standard deviation.

∗ Significant *P* value <.05.

In cost analysis for patients who did not undergo surgery, data showed that all non-CP minimum, maximum and average clinic visit cost were lower than that of CP as evidenced by significant *P* value of .00 (*P* < .05). When cumulated, total non-surgery clinic visit cost for non-CP group was lower at USD660.30 as compared to USD780.10 in CP group with a significant *P* value of .00 (*P* < .05) (Table 5).

**Table 5:** Cost analysis for non-surgery patients.

Cost	CP	SD	Non-CP	SD	Significance (*P* value)
Minimum non-surgery clinic visit (USD)	189.1	–	93.5		<.001^∗^
Maximum non-surgery clinic visit (USD)	198.6	–	171.2		<.001^∗^
Average non-surgery clinic visit (USD)	192.1	3.6	131.2	27.1	<.001^∗^
Total non-surgery clinic visit (USD)	780.1	4.7	660.3	194.9	<.001^∗^
Total cost for non-surgery group (USD)	780.1		660.3		

USD = US Dollar, CP = clinical pathway, SD = standard deviation.

∗ Significant *P* value <.05.

## 5 Discussion

Healthcare budget is finite. Hence, to streamline resources and still provide quality medical care, CP have been developed.^[3,15]^ In our study, we have developed a CP for CRSwNP to standardize medical care, optimize resources, reduce workload of medical personnel and to improve clinical outcomes.

A study by Schneider et al. correlated nasal polyp size to higher Sinonasal Outcome Test-20 (SNOT-20) scores. It revealed significantly higher impact on quality of life in CRSwNP patients as compared to those without polyps.^[16]^ In our study, the surgery group presented with higher grade of nasal polyp at first clinic visit (91.9% with grade 2 and 3 polyps) compared to non-surgery group (83.3% with grade 1 and 2 polyps). The findings correlate to patients with higher grade of polyps having a higher tendency to present earlier and undergo surgery as they are more symptomatic compared to those with lower grade polyps.

Randomized controlled trials of functional endoscopic sinus surgery (FESS) versus medical therapy for CRS have demonstrated no significant difference in treatment arms at end of 12 months as evidenced by improvements in nearly all parameters of Short Form 36 Health Survey and SNOT-20. This supports initial usage of optimal medical treatment and to proceed with surgery only in those who are refractory to treatment.^[17]^ In our study, mean duration from first clinic visit until operation date was 20.7 months. Based on CP, patients should have undergone surgery after 12 months of optimal medical treatment if patient's symptoms and nasal polyp size do not improve. With CP implementation, patients would have avoided delay in surgery.

Clarithromycin is a macrolide which has immunomodulatory and anti-inflammatory effect associated with polyp size reduction in both allergic and non-allergic patients.^[18]^ Macrolide is effective in treating neutrophil associated chronic rhinosinusitis without nasal polyposis but not effective in eosinophil predominant CRSwNP, characterized by eosinophilia, high serum immunoglobulin E levels, multiple polyposis, and asthma.^[19]^ However, in contrast to US or Europe, studies showed that in Eastern Asian populations, more than 50% of CRSwNP are non–eosinophil dominant and some show neutrophil dominant cell type.^[20,21]^ Therefore, certain subsets of Asian CRSwNP patients might benefit from macrolide treatment which is practiced in our center. Result showed that non-CP patients were given suboptimal treatment and medication is under-prescribed when compared to CP. Clarithromycin is given in just 36.5% of all clinic visits with average duration of 25.5 days which is less than 42 days required by CP.

Intranasal corticosteroid (INCS) function to reduce inflammation and eosinophilic infiltration thus reducing size of polyps which lead to improved nasal symptoms.^[22]^ CP recommends INCS dose of 2 sprays twice daily. However, this was prescribed correctly only 62.67% of the time showing under treatment. Suboptimal treatment is also evident in prescription of oral corticosteroids where it misses the CP's recommended dose of 14 days, 15 mg daily at each clinic visit. It was only prescribed in 20% of clinic visits with mean duration of 7.76 days. A study by Won et al on 47 patients with nasal polyposis given 20 mg of oral steroids administered daily for 14 days showed 62% had decreased polyp size of more than 25% and improved nasal symptoms as shown via decreased SNOT-20 scores.^[23]^

Nasal irrigation improves nasal symptoms through mucus clearance, removal of allergen, biofilm, and inflammatory mediators. CP recommends nasal irrigation for all CRSwNP patients, but our study showed under-prescription with irrigation given in only 15.6% of clinic visits. CP also recommends antihistamine in patients with allergy. Result showed prescription of antihistamines at 68.2%. However, data showed only 54.4% of patients had allergy and 56.3% had allergic rhinitis. This revealed that in a lot of cases, prescription of antihistamine was not justified.

Study by Panella et al^[15]^ showed usefulness of CP in efforts to promote standardization in treatment. CP on inguinal hernia repair showed that indiscriminate prescription of antibiotic prior to operation was no longer administered after implementation of CP. In CP of heart failure, the proportion of patients discharged with angiotensin converting enzyme inhibitor also increased post implementation of CP. In our study, we compared cost of non-CP group to the developed CP. In general, cost of preoperative visits for non-surgery and surgery groups were lower compared to CP. The lower cost is due to suboptimal treatment which is not in accordance with CP as seen in under-prescription of medication during follow-ups. In certain cases, medication was not prescribed, or patients given unsuitable medications. Visit cost was also affected by shorter intervals between clinic visits in non-CP group whereby the shorter duration of visit will result in lower cost per clinic visit compared to cost in CP due to reduced medication cost. Another contributing factor was patients defaulting appointments as patients will receive no medication during the period thus reducing total cost and the defaulted appointments will reduce top-down costing associated with clinic visits.

A study on CP of total hip replacement revealed significant changes in pre and post admission visits in which visit schedules were standardized post implementation of CP which reduced burden on patients.^[15]^ The reason for non-CP, surgery group's higher total preoperative visit cost was attributed to more frequent clinic visits with a mean interval of 2.1 months between visits and the lengthy duration of 20.67 months before patients underwent surgery. This is in contrast with CP which suggests a regular 4-monthly clinic visit interval. Therefore, with increased frequency of clinic visits, the cost is also higher due to the top-down costing (USD38.70 per visit) and activity-based costing involved in every clinic visit. Besides that, CP only calculates cost for 12 months visit duration as patients are expected to undergo operation by 12 months.

A meta-analysis study by Yong et al of 14 randomized controlled trial studies in China determined that use of CP in CRS patients undergoing surgery reduces cost, shortens hospital stay and increases patient satisfaction.^[24]^ Our results showed that surgery cost is higher for non-CP patients due to longer mean hospital stay of 3.98 days as compared to 3 days recommended in CP. This is due to additional top-down costing (USD342.90 per day) and activity-based costing such as medications. Besides that, mean surgery cost for CP is calculated based on patients undergoing either anterior FESS (USD190.40) or complete FESS (USD522.40). The significant difference in cost affects the calculated mean cost in CP.

Post-operatively for non-CP group, visit cost was higher compared to CP. One factor to consider is the 6-month post operation nasal polyp recurrence in 55.1% of patients which required additional medication. Postoperative cost in CP is calculated with assumption that patients have no nasal polyp recurrence post operation. Another reason for higher cost would be incorrect medication regimes given to non-CP patients. In summary, non-CP, non-surgery group has lower cost (USD660.30) compared to CP (USD780.10) mainly due to under treatment and patients defaulting visits. Meanwhile, total cost for non-CP surgery patients is higher (USD3600.20) compared to CP (USD2706) due to the longer follow-up duration, improper medication regime and longer hospital stay.

Study by Ragab et al^[17]^ had revealed no difference in sinonasal symptoms and outcomes after 12 months of optimal medical treatment or surgery. However, cases refractory to medical treatment should be subjected to surgery to see improvements in outcome. Our study showed that 88.9% of non-surgery patients still had grade 1 and 2 polyps at the end of 12 months follow-up period. Only 11.1% had resolution of polyp. This was due to suboptimal treatment which did not follow the CP. Surgery would be helpful to alleviate nasal symptoms in these group of non-surgery patients as can be seen from data showing 44.9% of surgery group had no polyp at 6 months post operation while those having polyps post operation were mostly low-grade polyp (44.9% grade 1).

### 5.1 Limitations of study

Limitations encountered in this study include suboptimal documentation by medical personnel in medical files of which they were excluded if found to be incomplete. Additionally, the use of electronic medical record is not practiced in our center and handwritten form of medical record is still used which can lead to missing data if pages are missing or handwritings are faded. Variables such as age and race could potentially be confounders which we can investigate further in future studies. Results of CRSwNP patients with history of refractory polyposis after multiple surgeries were also cautiously analyzed as it is associated with high risk of recurrence and worse outcomes compared to patients who have not undergone surgery before. These CRSwNP patients who are recalcitrant to treatment can be due to coexisting fungal or viral infection. It can also be attributed to specific CRSwNP phenotypes of eosinophilic or neutrophilic subtypes. However, in the setting of this study, we only study the general CRSwNP sample population. We are not able to specifically investigate the histopathology or do phenotyping for the patients as this is not standard practice during treatment and also due to cost. Since this study was done in a university hospital, there were also limitations where patients involved in other clinical studies had to be excluded.

## 6 Conclusion

This study showed that non-CP patients were being undertreated compared to CP. Non-CP patients were also subjected to unnecessary cost and delays. We were also able to identify aspects of clinical practice which resulted in wastage and unnecessary burden to our healthcare system. This study result would enable the development of a written CP by allowing us to fine tune the various aspects of CP which could then be applied to our clinical practice in the future.

## Acknowledgments

I would like to acknowledge my supervisor and co-supervisors who have contributed to this research paper. I would also like to thank all the related staff from various departments who have assisted me in their various capabilities in ensuring the completion of this research.

## Author contributions

**Conceptualization:** Timothy Wong, Salina Husain, Aniza Ismail, Farah Dayana Zahedi.

**Data curation:** Timothy Wong, Salina Husain, Aniza Ismail.

**Formal analysis:** Timothy Wong, Salina Husain, Aniza Ismail, Syed Mohamed Aljunid, Amrizal Muhammad Nur.

**Funding acquisition:** Timothy Wong.

**Investigation:** Timothy Wong, Aniza Ismail.

**Methodology:** Timothy Wong, Salina Husain, Aniza Ismail.

**Project administration:** Timothy Wong, Salina Husain, Aniza Ismail, Farah Dayana Zahedi.

**Resources:** Timothy Wong.

**Software:** Timothy Wong.

**Supervision:** Salina Husain, Aniza Ismail, Farah Dayana Zahedi, Syed Mohamed Aljunid, Amrizal Muhammad Nur.

**Validation:** Salina Husain, Aniza Ismail, Farah Dayana Zahedi, Syed Mohamed Aljunid, Amrizal Muhammad Nur.

**Visualization:** Timothy Wong, Aniza Ismail.

**Writing – original draft:** Timothy Wong.

**Writing – review & editing:** Timothy Wong, Salina Husain, Aniza Ismail.
